# Virulent and multidrug-resistant *Aeromonas* in aquatic environments of Kerala, India: potential risks to fish and humans

**DOI:** 10.1007/s42770-024-01601-w

**Published:** 2025-01-14

**Authors:** Vandan Nagar, Farhat Ansari, Murugadas Vaiyapuri, Toms C. Joseph

**Affiliations:** 1 Food Technology Division, Bhabha Atomic Research Centre, Trombay, Mumbai, 400085 India; 2 Homi Bhabha National Institute, Anushaktinagar, Mumbai, 400094 India; 3 ICAR-Central Institute of Fisheries Technology (ICAR-CIFT), Willingdon Island, Cochin, Kerala 682029 India

**Keywords:** Aeromoniasis, Water bodies, Virulent determinants, Antibiotic resistance, Biofilm, Integrons

## Abstract

*Aeromonas* inhabit diverse aquatic habitats and are recognized as both opportunistic and primary pathogens of fish and humans. This study delineates the biochemical and *gyrB* sequence-based molecular identification of 14 *Aeromonas* strains isolated from aquatic environments in Kerala, India, identifying them as *A. dhakensis* (50%), *A. hydrophila* (28.6%), and *A. jandaei* (21.4%). These strains exhibit a high prevalence of virulence genes (*act*, *flaA*, *ser*, *gcat*, *lip*, and *ela*) implicated in pathogenesis in both fish and humans. These findings underline the emergence of *A. dhakensis*, often misidentified as *A. hydrophila*, as a potential pathogen, highlighting the necessity for comprehensive identification methods. Significantly, all strains demonstrated beta-hemolysis and moderate to strong biofilm formation, enhancing their infectivity potential. Moreover, all isolates exhibited multidrug resistance, with a multiple antimicrobial resistance (MAR) index ranging from 0.39 to 0.56, and a significant presence of class 1 (500–1100 bp) and class 2 (250–700 bp) integrons, indicating their potential risk to both fish and human populations. Our results underscore the role of aquatic environment as a repository for virulent and multidrug-resistant *Aeromonas* spp., emphasizing the imperative for prudent antimicrobial usage and regular monitoring of antimicrobial resistance (AMR) in these environments.

**Supplementary Information:**

The online version contains supplementary material available at 10.1007/s42770-024-01601-w.

## Introduction

The genus *Aeromonas*, comprising 36 gram-negative aquatic species, represents a diverse group with notable implications for aquatic ecosystems and public health. These species are significant fish pathogens, causing diseases like haemorrhagic septicemia and furunculosis, leading to large-scale outbreaks in aquaculture farms [1]. Additionally, they are recognized as emerging opportunistic pathogens, transitioning from environmental sources to humans and causing various intestinal and extra-intestinal infections [2]. *Aeromonas* plays a critical role in the One Health concept, being associated with economic losses in aquaculture, the spread of antibiotic resistance genes, and infections related to leech therapy in humans [3].

*Aeromonas* species are ubiquitous, found in aquatic environments, soil, and food, posing significant public health challenges [4]. Among them, *A. hydrophila*, *A. dhakensis*, *A. caviae*, and *A. veronii* (biovar *veronii* and *sobria*) are responsible for over 95% of human intestinal infections caused by *Aeromonas* [5]. However, *A. dhakensis* is often misidentified as *A. hydrophila*, leading to an underestimation of its cases [6, 7]. Research shows that *A. dhakensis* is predominant in tropical and subtropical regions and exhibits greater virulence than *A. veronii*, *A. caviae*, and *A. hydrophila* [5, 8]. Recently, virulent and antibiotic-resistant *A. dhakensis* strains have been increasingly reported from various environmental sources, causing numerous infections in fish and humans [6, 9]. Species-level identification of *Aeromonas* is complex and challenging due to the lack of standardized phenotypic tests, discrepancies between phenotypic and genotypic methods, genetic heterogeneity, and the increasing number of new species [4]. Recent studies have utilized housekeeping genes (*gyrB*, *rpoD*, and *rpoB*) as molecular markers for precise species-level identification and to establish phylogenetic relationships [2, 6, 10].

The virulence potential of *Aeromonas* is complex, dependent on host susceptibility, and not fully understood [3]. Various virulence factors, including cell-associated structural components, extracellular products, toxins, and biofilm formation, enable *Aeromonas* cells to colonize and destroy host cells [11]. In recent years, antibiotic resistance among *Aeromonas* strains has become a significant public health concern, particularly in developing countries where the use of antibiotics is often unregulated and uncontrolled [5]. Pathogens affecting humans, animals, and the environment face the dual challenges of increased virulence determinants and growing antimicrobial resistance. Therefore, this study aimed to identify *Aeromonas* strains, isolated from aquatic environments of Kerala, India and examine their pathogenicity, biofilm formation, and resistance potential.

## Materials and methods

### *Aeromonas* cultures and biochemical identification

Fourteen presumptive *Aeromonas* strains, previously isolated from both healthy and moribund finfish exhibiting symptoms such as exophthalmia, abdominal distention, haemorrhagic spots, necrotic fin rot, and ragged fins, were collected from aquaculture farms and river water samples in Ernakulam and Thrissur, Kerala, India (Suppl Table 1). The affected animals, including species such as Catla (*Catla catla*), Rohu (*Labeo rohita*), and Gourami (family *Osphronemidae*), were brought to the laboratory by farmers. These farms utilized traditional intensive freshwater aquaculture ponds, either for food fish production or for rearing ornamental fish. The isolates were identified to the species level using comprehensive biochemical tests [12–14]. The strains’ hemolytic activity was assessed on blood agar plates (HiMedia, India) incubated at 30 °C for 18 h [15].

### Molecular Identification and phylogenetic analysis

All biochemically identified *Aeromonas* species were confirmed through *gyrB* (~ 1100 bp) gene sequencing, following the primers and procedures of Soler et al. [16]. The amplified products were purified, sequenced, and compared against GenBank database entries using the BLASTN program (www.ncbi.nih.gov/BLAST/). These sequences were then uploaded to GenBank (Accession numbers: OQ743456-64, OQ789562-65, and OQ920270). The *gyrB* gene sequences were aligned, and sequence similarities were estimated for a 920 base segment (positions 852,606–853,528, according to *E. coli* ATCC 25922 numbering, NZ_CP009072). A phylogenetic tree was constructed using the UPGMA method and Kimura two-parameter model in MEGA 11 [17].

### Virulence genes

Virulence genes, including aerolysin (*aer*), cytotoxic enterotoxin (*act*), hemolysin (*hly*), lipase (*lip*), elastase (*ahyB*), glycerophospholipid cholesterol acyltransferase (*gcat*), cytotonic enterotoxins (*ast*, *alt*), serine protease (*ser*), polar flagella (*fla*), and lateral flagella (*lafA*), were amplified from chromosomal DNA using previously described primers and PCR conditions [18, 19].

### Biofilm formation and analysis

Biofilm formation was evaluated by culturing various *Aeromonas* strains in TSB within 96-well flat-bottomed microtiter plates (Falcon, BD Biosciences, NJ, USA) and quantified using a modified crystal violet (CV) assay [19]. The biofilm-forming ability was classified using the specific biofilm formation (SBF) index, calculated for each isolate by normalizing biofilm accumulation (OD_570nm_) relative to cell growth (OD_600nm_) with the formula SBF = (B - NC)/G, where B represents the OD_570nm_ of the attached and stained bacteria, NC is the OD_570nm_ of the control wells containing bacteria-free medium, and G is the OD_600nm_ of cell growth in the medium [20]. The extent of biofilm formation was categorized into three groups: no biofilm (SBF < 0.1), weak (0.1 ≤ SBF ≤ 0.5), moderate (0.5 ≤ SBF ≤ 1), or strong (SBF > 1) [21].

### Antimicrobial susceptibility and integron analysis

The antimicrobial resistance patterns of these strains were determined against 18 commonly used antibiotics from 11 classes, using the disc diffusion method on Mueller-Hinton Agar, as specified by the Clinical and Laboratory Standards Institute (CLSI) [22]. Resistance profiles were assigned based on zone diameters according to CLSI breakpoints [23]. Multi-drug resistance and multiple antibiotic resistance (MAR) indices were calculated following Magiorakos et al. [24] and Krumperman [25], respectively. The MAR index for a single isolate is calculated as the ratio of *a* to *b*, where *a* is the number of antibiotics the isolate is resistant to, and *b* is the total number of antibiotics it has been exposed to. The presence of class 1 and class 2 integrons was assessed under conditions described by Nagar et al. [19].

## Results and discussion

### Identification of *Aeromonas* from aquatic environment

Fourteen presumptive *Aeromonas* isolates were identified at the genus level based on biochemical tests (positive oxidase, nitrate reductase reactions, catalase; fermentation of D-glucose and trehalose; utilization of dulcitol, D-arabitol, erythritol and xylose; and resistance to vibriostatic agent O/129), as described by Abbott et al. [12] and Nagar et al. [10]. Based on the additional biochemical characteristics: aesculin hydrolysis, and acid production from L-arabinose, urocanic acid, salicin, sucrose, D-cellobiose and L-fucose [13, 14], they were further identified up to the species level (Table 1).

**Table 1:** Phenotypic and genetic identification, and integrons of 14 *Aeromonas* strains isolated from aquatic environment of Kerala

Strain	Source	Taxonomic identification (species name) based on		NCBI Acc. No.	Class 1 integron (bp)	Class 2 integron (bp)
		**Biochemical tests**	***gyrB gene***			
A1	River_ Ernakulam	*A. dhakensis*	*A. dhakensis*	OQ743456	650, 1100	650
A2	River_ Ernakulam	*A. dhakensis*	*A. dhakensis*	OQ743457	650, 1100	-
A3	River_ Ernakulam	*A. hydrophila*	*A. hydrophila*	OQ920270	-	-
A4	River_ Ernakulam	*A. dhakensis*	*A. dhakensis*	OQ789562	1100	-
A5	River_ Ernakulam	*A. dhakensis*	*A. dhakensis*	OQ789563	500	650
A6	River_Thrissur	*A. dhakensis*	*A. dhakensis*	OQ789564	500	650
A7	River_Thrissur	*A. dhakensis*	*A. dhakensis*	OQ743458	1100	-
A8	River_Thrissur	*A. hydrophila*	*A. hydrophila*	OQ743459	650	650
A9	River_Thrissur	*A. dhakensis*	*A. dhakensis*	OQ789565	550	700
M14	Catla, Aquaculture pond, Thrissur	*A. hydrophila*	*A. hydrophila*	OQ743460	-	-
M22	Rohu, Aquaculture pond, Thrissur	*A. hydrophila*	*A. hydrophila*	OQ743461	-	250
N14	Catla, Aquaculture pond, Ernakulam	*A. jandaei*	*A. jandaei*	OQ743463	800	-
N46	Rohu, Aquaculture pond, Ernakulam	*A. jandaei*	*A. jandaei*	OQ743464	-	-
P31	Gourami, Aquaculture pond, Ernakulam	*A. jandaei*	*A. jandaei*	OQ743462	850	-

Relying solely on morphological and biochemical characteristics for *Aeromonas* identification is often contentious and unreliable, leading to potential misidentifications [5]. Therefore, the *gyrB* gene, which has higher discriminatory power for phylogenetic analysis [16], was employed for species-level identification of the biochemically characterized *Aeromonas* strains. Partial *gyrB* gene sequences were submitted to GenBank at NCBI (Accession numbers: OQ743456-64, OQ789562-65, and OQ920270). Based on biochemical tests and *gyrB* gene sequences, these *Aeromonas* strains were identified and confirmed as *A. dhakensis* (50%), *A. hydrophila* (28.6%), and *A. jandaei* (21.4%). Initially misidentified as *A. hydrophila* in 2002, *A. dhakensis* was later recognized as a distinct species in 2013 [8]. Since then, *A. dhakensis* has been isolated from diverse sources across different countries with varying frequencies [6, 7, 9].

**Fig. 1:**
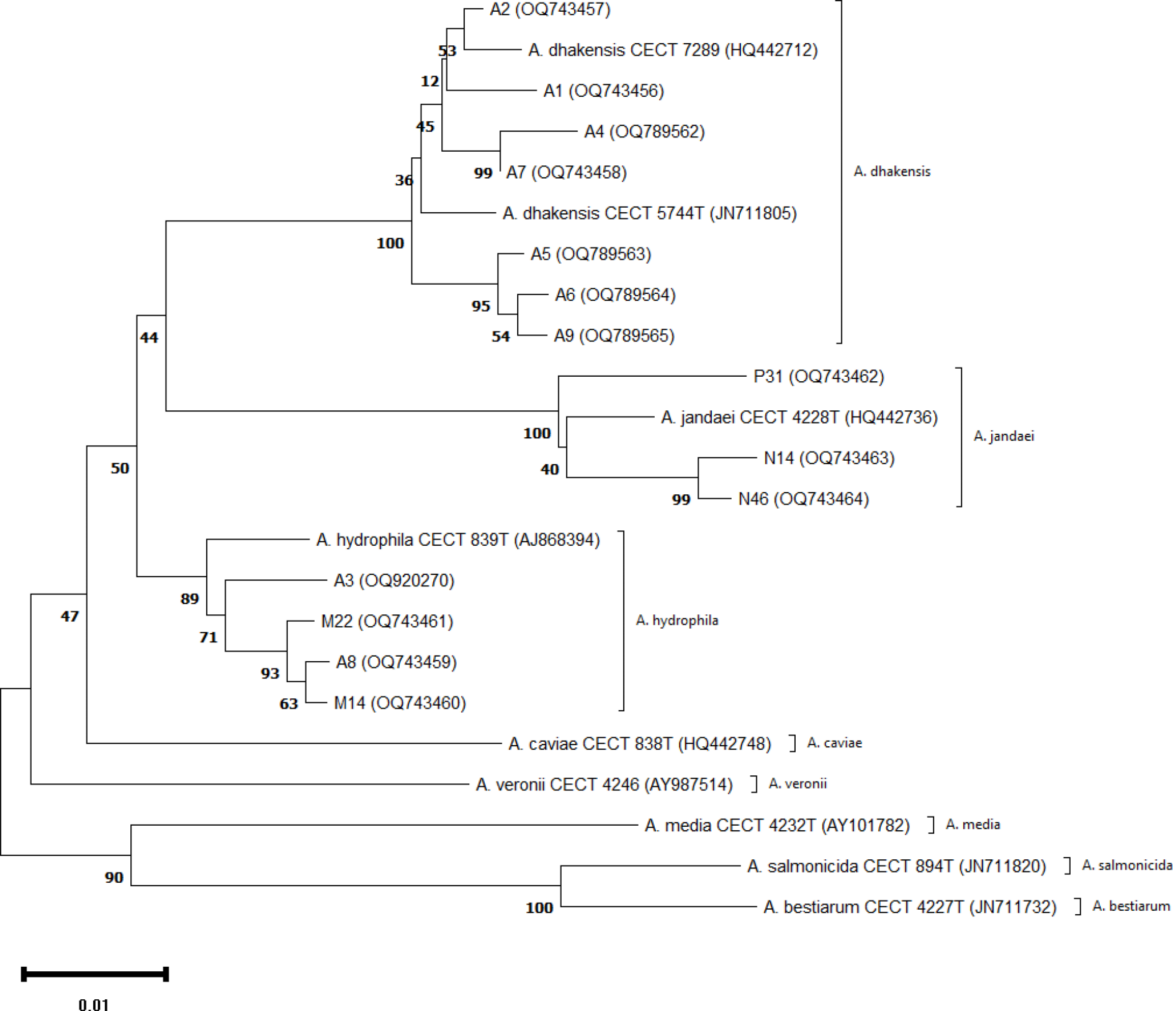
Unrooted phylogenetic tree (UPGMA) of *Aeromonas* isolates and other known *Aeromonas* species based on the *gyrB* gene sequences. CECT numbers indicate the Spanish Type culture collection numbers of the *Aeromonas* reference strains. Numbers in the parenthesis represent the GenBank accession numbers. Numbers shown next to each node indicate bootstrap values (percentage of 1,000 replicates). The bar indicates a sequence divergence

Pairwise and mean distances of aligned *gyrB* sequences from these strains were estimated for a continuous stretch of 920 bases (positions 852,606–853,528 according to *E. coli* ATCC 25922 numbering, NZ_CP009072) using ClustalW. The sequence homology among all *Aeromonas* strains for the *gyrB* gene ranged from 89.91 to 99.67%, representing 3 to 93 nucleotide variations. The mean sequence similarity, serving as a measure of discriminatory capability, was determined to be 95.0%, comparable to the 92.2% for the *gyrB* gene of *Aeromonas* strains described by Soler et al. [16]. The alignment revealed a total of 173 variable positions (18.8% of the sequenced fragment), along with a single triplet (ACA) insertion in *A. salmonicida* (CECT 894^T^) and *A. bestiarum* (CECT 4227^T^). Intra-species nucleotide substitution rates, determined by calculating mean distance within each species, ranged from 1.18% in *A. hydrophila* to 1.93% in *A. jandaei*. A phylogenetic tree constructed using these genetic matrices showed significant divergence among all *Aeromonas* species, with consistent clustering patterns between the investigated strains and their type or reference strains (Fig. 1).

This study reinforces the importance of a combined phenotypic and genotypic approach for reliable taxonomic classification of *Aeromonas* from diverse samples, as previously reported [2, 9]. *A. dhakensis*, *A. hydrophila*, and *A. jandaei* have been reported from fish and aquatic environments [11], as well as clinical and outbreak samples [6, 8]. The rising prevalence of *Aeromonas* species, known pathogens for both humans and aquatic animals, in aquatic environments raises concerns about increased infections in both populations, especially given the growing demand for seafood and the potential for fish-to-human transmission.

### Virulence genes analysis

Virulence factors in *Aeromonas* strains contribute to their pathogenesis and transmission. All aquatic *Aeromonas* strains in this study carried the genes *act* (232 bp), *ser* (350 bp), *gcat* (237 bp), *ahyB* (540 bp), and *lip* (390 bp) (Table 2). The *aer* (252 bp) and *hlyA* (597 bp) genes were present in all *A. dhakensis* and *A. hydrophila* strains, but only 33.3% and none of the *A. jandaei* strains, respectively (Table 2). Cytotonic enterotoxins, *alt* and *ast*, were found in 78.6% and 64.3% of the strains, respectively (Table 2). Both genes were highly prevalent in *A. dhakensis* and *A. hydrophila* strains (> 70%), but only 33.3% and none of the *A. jandaei* strains, respectively. Structural genes for polar flagella (*fla*) and lateral flagella (*lafA*) were present in 100% and 28.6% of these *Aeromonas* strains, respectively (Table 2). All *A. dhakensis* and *A. hydrophila* strains harbored *act*, *aer*, *hlyA*, and *fla* genes, while *ast*, *hlyA*, and *lafA* genes were absent in all *A. jandaei* strains. Recently, *Aeromonas* strains with various virulence genes have been reported from water [9], aquaculture farms [2], and clinical samples [6]. In this study, all strains exhibited distinct β-hemolysis zones on blood agar plates, indicating potential pathogenicity (Table 2). Hoel et al. [26] documented β-hemolysis in 91% of food strains.

**Table 2:** Distribution of genotypic virulence markers and β-hemolysis in *Aeromonas* strains

*Aeromonas* spp.	Cytotoxic enterotoxin	Cytotonic enterotoxins		Aerolysin / haemolysin genes		Flagellar genes		Serine protease	GCAT	Lipase (390)	Ela (540)	β-hemolysis
	*act* (232)	Heat-stable *ast* (331)	Heat-labile *alt* (442)	*aer* (252)	*hlyA* (597)	*lafA* (550)	*flaA* (608)	*ser* (350)	*gcat *(237)	*lip *(390)	*ela* (540)	
*A. dhakensis* (*n* = 7)	7 (100)	5 (71.4)	7 (100)	7 (100)	7 (100)	2 (28.6)	7 (100)	7 (100)	7 (100)	7 (100)	7 (100)	7 (100)
*A. hydrophila* (*n* = 4)	4 (100)	4 (100)	3 (75)	4 (100)	4 (100)	2 (50)	4 (100)	4 (100)	4 (100)	4 (100)	4 (100)	4 (100)
*A. jandaei* (*n* = 3)	3 (100)	0 (0)	1 (33.3)	1 (33.3)	0 (0)	0 (0)	3 (100)	3 (100)	3 (100)	3 (100)	3 (100)	3 (100)
Total (*n* = 14)	14 (100)	9 (64.3)	11 (78.6)	12 (85.7)	11 (78.6)	4 (28.6)	14 (100)	14 (100)	14 (100)	14 (100)	14 (100)	14 (100)

### Biofilm

**Fig. 2:**
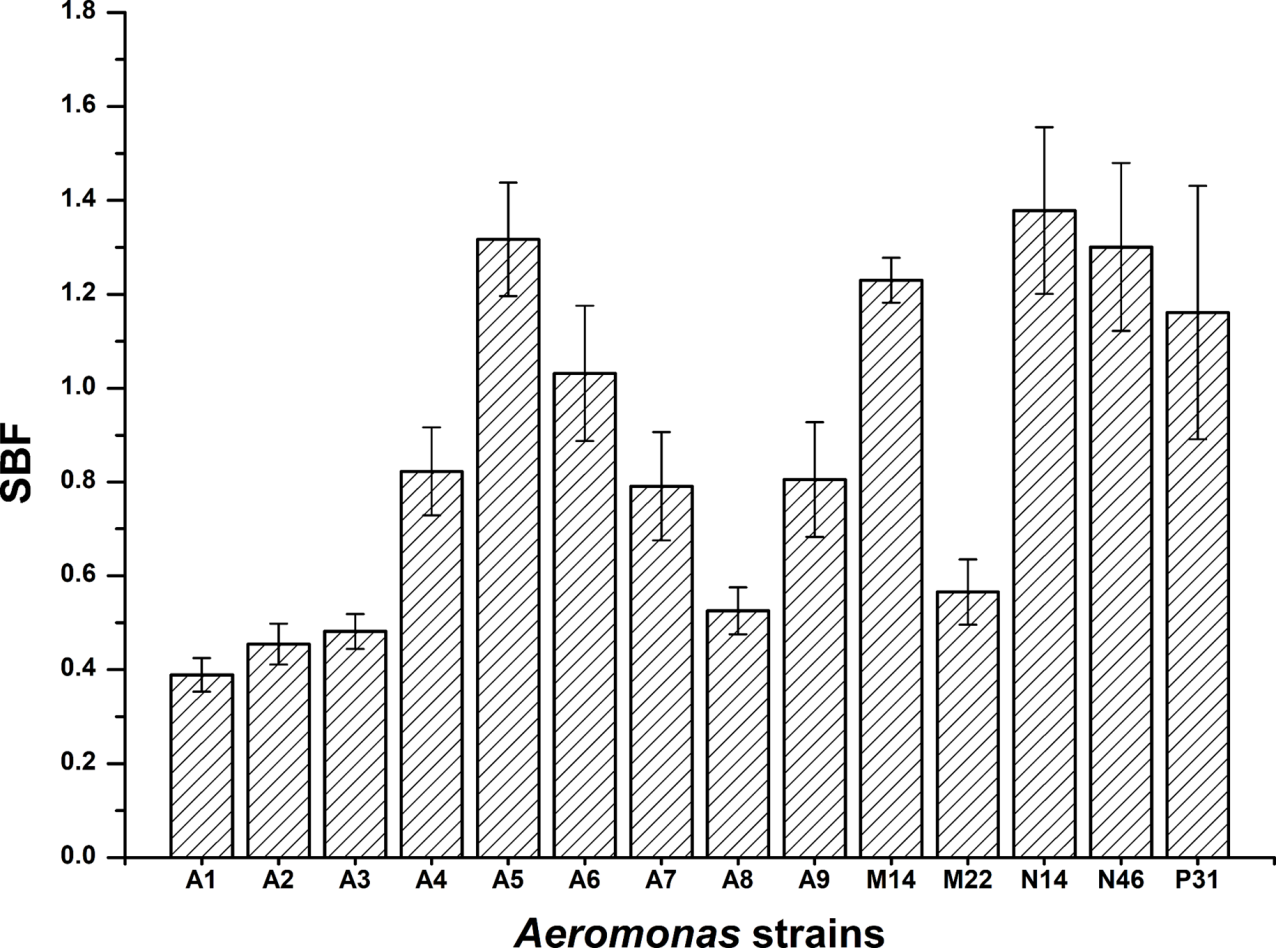
Biofilm formation by *Aeromonas* strains in TSB 30 °C. Bars represent average SBF values and standard errors

Biofilm formation enhances bacterial survival and virulence. *Aeromonas* can adhere to and colonize both biotic and abiotic surfaces [1]. Biofilm formation was measured using the SBF index, which incorporates bacterial growth rate (OD_600nm_) for consistent categorization, as described by Naves et al. [20]. In this study, biofilm formation by 14 *Aeromonas* strains in TSB ranged from 0.39 to 1.38. The strains were categorized as weak (*n* = 3, 21.4%), moderate (*n* = 5, 35.7%), and strong (*n* = 6, 42.9%) biofilm producers (Fig. 2). All *A. jandaei* strains were strong biofilm producers, while *A. dhakensis* and *A. hydrophila* showed no clear pattern. *Aeromonas* strains from fish produced moderate to strong biofilms, whereas those from water samples were predominantly weak to moderate producers. This aligns with research by Chenia and Duma [27], showing that most *Aeromonas* strains from freshwater fish and seafood produce moderate to strong biofilms, while strains from estuarine and river waters typically produce weak biofilms [28]. Chen et al. [8] reported that clinical *A. dhakensis* strains form stronger biofilms compared to *A. hydrophila* strains in Taiwan.

### Antimicrobial susceptibility

The antimicrobial susceptibility of 14 confirmed *Aeromonas* strains was evaluated against 18 antibiotics to create an antibiogram profile. All strains were resistant to ampicillin, cephalothin, imipenem, ampicillin/sulbactam, aztreonam, piperacillin-tazobactam, and clindamycin, but were sensitive to gentamicin, co-trimoxazole, amikacin, chloramphenicol, nitrofuran, and ciprofloxacin (Table 3). Notably, strains from water samples in Ernakulam and Thrissur showed resistance to ceftriaxone and nalidixic acid, whereas strains from fish samples were sensitive to these antibiotics. This antimicrobial resistance (AMR) pattern aligns with findings by Dubey et al. [2].

**Table 3:** Percentage antimicrobial resistance of *Aeromonas* spp. isolated from aquatic environment of Kerala

Antibiotic (concentration, µg)	*A. dhakensis* (*n* = 7)			*A. hydrophila* (*n* = 4)			*A. jandaei* (*n* = 3)			Resistant strains (%)
	*R*	I	S	*R*	I	S	*R*	I	S	
Ampicillin (10)	100			100			100			100
Ampicillin/Sulbactam (10/10)	100			100			100			100
Piperacillin/Tazobactam (100/10)	100			100			100			100
Tetracycline (30)	71.4		28.6	25		75			100	42.9
Gentamicin (10)			100			100			100	-
Streptomycin (10)		14.3	85.7			100			100	-
Kanamycin (30)		14.3	85.7			100			100	-
Amikacin (30)			100			100			100	-
Trimethoprim/sulfamethoxazole (25)			100			100			100	-
Chloramphenicol (30)			100			100			100	-
Cephalothin (30)	100			100			100			100
Ceftriaxone (30)	100			50	25	25			100	64.3
Imipenem (10)	100			100			100			100
Nitrofurantoin (30)			100			100			100	-
Aztreonam (30)	100			100			100			100
Ciprofloxacin (5)			100			100			100	-
Nalidixic Acid (30)	71.4		28.6	25		75			100	42.9
Clindamycin (2)	100			100			100			100

*Aeromonas*, a ubiquitous water-borne organism, easily acquires and exchanges AMR genes, making it a recognized reservoir of antibiotic resistance genes (ARGs) [3]. Water environments host diverse microbial communities and ARG reservoirs. Overuse of antibiotics and inadequate sanitation expose these communities to external ARGs, accelerating their acquisition and dissemination. In this study, 43% of strains were resistant to tetracycline, although this was lower than in recent studies by Jacobs and Chenia [29].

Multidrug resistance, defined as resistance to at least one antimicrobial agent from three or more different classes [24], was observed in all *Aeromonas* strains. Six strains (A4, A5, A6, A7, A8, and A9) resisted six antibiotic classes: penicillin and its derivatives, tetracycline, first-generation cephalosporins, penems, monobactams, and fluoroquinolones. Eight strains (A1, A2, A3, M14, M22, P31, N14, and N46) resisted four classes: penicillin and its derivatives, first-generation cephalosporins, penems, and monobactams. The prevalence of AMR microorganisms is rising and is expected to become a major public health concern.

Our findings corroborate earlier research on the emergence of multidrug-resistant *Aeromonas* strains in river water, fish, food, and clinical settings [5, 29, 30]. The Multiple Antibiotic Resistance (MAR) index, a valuable risk assessment tool, indicated MAR values between 0.39 and 0.56 (Suppl Table 2) for *Aeromonas* strains in this study, with all values exceeding 0.2. This suggests contamination from sources with frequent antimicrobial use, indicating a high-risk environment. The MAR index range observed is consistent with previous research [28, 29], further implying potential antimicrobial contamination in the surveyed aquatic environments. To control antimicrobial-resistant *Aeromonas* in these environments, farmers and veterinary teams should prioritize strong biosecurity measures, such as maintaining water quality and reducing animal stress, while incorporating alternatives like phage therapy, probiotics, vaccines, plant-based antimicrobials, and silver nanoparticles. Phage therapy, in particular, offers a targeted approach to combat resistance, while responsible antibiotic use, effective water management, and staff education are essential for the long-term health and sustainability of aquaculture systems.

### Integron analysis

Class 1 integrons were identified in 71.4% of the *Aeromonas* aquatic strains, with variable region sizes ranging from 500 to 1100 bp; the remaining 4 strains showed no amplification (Table 1). These results are consistent with previous research showing 21.7% of *Aeromonas* strains from ornamental freshwater fish farms carrying class 1 integrons [9]. Additionally, 42.9% of strains exhibited class 2 integrons (250–700 bp) (Table 1), aligning with findings by Jacobs and Chenia [29] and Nagar et al. [19], who reported class 2 integrons in 27% of aquaculture and 50% of food strains, respectively.

Integrons, mobile genetic elements, facilitate the integration and expression of diverse gene cassettes, including antibiotic resistance genes. They play a significant role in promoting antibiotic resistance within environmental bacterial populations via horizontal gene transfer [3]. In *Aeromonas* strains, integrons could potentially enhance the transfer of multiple antibiotic resistance genes among environmental microorganisms.

## Conclusions

The study identified diverse *Aeromonas* species, including potentially pathogenic strains, in water and fish samples from Kerala, India. By combining *gyrB* analysis with biochemical tests, accurate species-level identification was achieved, highlighting the importance of these methods in differentiating *Aeromonas* species. The high occurrence of *A. dhakensis* raises significant concerns for India’s freshwater and aquaculture ecosystems. The presence of pathogenic *Aeromonas* strains in Indian aquaculture underscores the critical need for regular surveillance and stringent antibiotic controls. Their varied biofilm-forming abilities and widespread antibiotic resistance, including multidrug resistance, suggest that aquatic environments may act as reservoirs for resistant bacteria, potentially promoting the emergence of more virulent strains due to antibiotic misuse. Responsible antibiotic use is crucial for the sustainability of Indian aquaculture and the health of its ecosystems. This study focused on characterizing *Aeromonas* strains for therapeutic phage recovery in the treatment of aeromoniasis, revealing diverse strains with varying levels of virulence and resistance. Future work will explore bacteriophages and bacteriophage-derived proteins for their potential therapeutic applications in combating *Aeromonas* infections.

## Electronic supplementary material

Below is the link to the electronic supplementary material.

Supplementary Material 1

## Data Availability

All data included in this study are available upon request. Partial *gyrB* gene sequences of *Aeromonas* strains have been submitted to GenBank at NCBI (Accession numbers: OQ743456-64, OQ789562-65 and OQ920270).
