# Supplementary material for: Virulent and multidrug-resistant Aeromonas in aquatic environments of Kerala, India: potential risks to fish and humans
Source: Braz J Microbiol. 2025 Jan 14;56(1):303–11. doi: 10.1007/s42770-024-01601-w (PMC11885762; doi:10.1007/s42770-024-01601-w)
Supplement: Supplementary file 1 — Supplementary Material 1 [file 42770_2024_1601_MOESM1_ESM.docx]

**Supplementary data**

**Virulent and Multidrug-Resistant *Aeromonas* in Aquatic Environments of Kerala, India: Potential Risks to Fish and Humans**

**Journal: Brazilian Journal of Microbiology**

Vandan Nagar^a,b^*, Farhat Ansari^a,b^, Murugadas Vaiyapuri^c^ and Toms C. Joseph^c^

^a^Food Technology Division, Bhabha Atomic Research Centre, Trombay, Mumbai, 400085, India

^b^Homi Bhabha National Institute, Anushaktinagar, Mumbai, 400094, India

^c^ICAR-Central Institute of Fisheries Technology (ICAR-CIFT), Willingdon Island, Cochin, 682029, Kerala, India

*Corresponding author

Dr. Vandan Nagar,

Food Technology Division, Bhabha Atomic Research Centre, Mumbai 400 085, India.

Tel: 91-22-25593961/ 25590577

Fax: 91-22-25505151

Email: vnagar@barc.gov.in

ORCID: 0000-0001-6556-8068

Supplementary Table 1: Details of *Aeromonas* spp. isolated from previous studies of aquaculture farms of Kerala, India

| Source | No. of samples | No. of *Aeromonas* positive samples (%) | *A. dhakensis^a^* | *A. hydrophila^a^* | *A. jandaei^a^* |
| --- | --- | --- | --- | --- | --- |
| Water_Ernakulam | 7 | 5 (71.4%) | 4 | 1 | - |
| Water_Thrissur | 7 | 4 (57.1%) | 3 | 1 | - |
| Fish_ Ernakulam | 5 | 3 (60.0%) | - | - | 3 |
| Fish_ Thrissur | 5 | 2 (40.0%) | - | 2 | - |
| Total | 24 | 14 (58.3%) | 7 | 4 | 3 |

*^a^* Number of strains isolated

Supplementary Table 2: The percentage occurrence of multiple antibiotic resistance (MAR) index of *Aeromonas* strains

| MAR index | *Aeromonas* species | | |
| --- | --- | --- | --- |
|  | *A. dhakensis* (n=7) | *A. hydrophila* (n=4) | *A. jandaei* (n=3) |
| 0.4 | 0 | 2 (50%) | 3 (100%) |
| 0.45 | 2 (28.6%) | 1 (25%) | 0 |
| 0.5 | 0 | 0 | 0 |
| 0.55 | 0 | 0 | 0 |
| 0.6 | 5 (71.4%) | 1 (25%) | 0 |
